# Next generation sequencing of *RB1*gene for the molecular diagnosis of ethnic minority with retinoblastoma in Yunnan

**DOI:** 10.1186/s12881-020-01150-7

**Published:** 2020-11-23

**Authors:** Zhen Zhang, Yi-shuang Xiao, Ru Shen, Hong-chao Jiang, Li Tan, Ren-qiu Li, Xiao-hong Yang, Huai-yu Gu, Wen-Ji He, Jing Ma

**Affiliations:** 1 Key Laboratory of Childrenʼs Major Disease Research, and Yunnan Institute of Pediatrics, Kunming Childrenʼs Hospital, Kunming Medical University, Kunming, Yunnan 650228 PR China; 2 Department of Human Anatomy, Zhongshan School of Medicine, Sun Yat-Sen University, Guangzhou, Guangdong 510080 PR China; 3 Department of Ophthalmology, Kunming Children’s Hospital, Kunming Medical University, Kunming, Yunnan 650228 PR China; 4 Kunming Children’s Hospital, Kunming Medical University, Kunming, Yunnan 650228 PR China; 5 Department of Otolaryngology-Head Neck Surgery, Kunming Children’s Hospital, Kunming Medical University,, Kunming, Yunnan 650228 PR China

**Keywords:** Retinoblastoma, Mutations, Targeted next generation sequencing, Genetic forms

## Abstract

**Background:**

Retinoblastoma is a rare intraocular malignancy and typically initiated by inactivating biallelic mutations of *RB1* gene. Each year, ~ 8000 children worldwide are diagnosed for retinoblastoma. In high-income countries, patient survival is over 95% while low-income countries is ~ 30%.If disease is diagnosed early and treated in centers specializing in retinoblastoma, the survival might exceed 95% and many eyes could be safely treated and support a lifetime of good vision. In China, approximate 1100 newly diagnosed cases are expected annually and 28 hospitals covering 25 provinces established centers classified by expertise and resources for better treatment options and follow-up. Comparing with other province of eastern China, Yunnan province is remote geographically. This might result that healthcare staff have low awareness of the role of genetic testing in management and screening in families.

**Methods:**

The patients with retinoblastoma were selected in Yunnan. DNA from blood was used for targeted gene sequencing. Then, an in-house bioinformatics pipeline was done to detect both single nucleotide variants and small insertions/deletions. The pathogenic mutations were identified and further confirmed by conventional methods and cosegregation in families.

**Results:**

Using our approach, targeted next generation sequencing was used to detect the mutation of these 12 probands. Bioinformatic predictions showed that nine mutations were found in our study and four were novel pathogenic variants in these nine mutations.

**Conclusions:**

It’s the first report to describe RB1 mutations in Yunnan children with retinoblastoma. This study would improve role of genetic testing for management and family screening.

**Supplementary Information:**

The online version contains supplementary material available at 10.1186/s12881-020-01150-7.

## Background

Retinoblastoma (RB, OMIM#180200) is a rare malignant tumor which rapidly develops from the immature cells of a retina and occurs in infancy or in children, usually before the age of 5 years (about 2/3 children below the age of 2 years, about 95% children below the age of 5 years) [1, 2].

To explain mechanisms of RB, Knudson proposed the famous “two-hit hypothesis”. A germline mutation (‘first hit’, M1) and an acquired somatic mutation (‘second hit’, M2) arise heritable retinoblastomas; two somatic mutations presented in the same transformation suppressor gene in a susceptible cell, result from non-heritable retinoblastomas [3]. In some patients, Chromosomal deletions pointed to a chromosome (chr.) 13q14 locus. In 70% of retinoblastoma tumors, the heterozygosity of chr. 13q14 polymorphic loss suggested the second hit involved the same locus [4].

The first cloned gene of tumor suppressor is retinoblastoma 1 (*Rb*1) gene. The protein which is encoded by this gene is a cell cycle negative regulator. To maintain the overall chromatin structure, this encoded protein stabilizes constitutive heterochromatin. Defect in *Rb*1 is a cause of childhood cancer Rb, osteogenic sarcoma, and bladder cancer [5].

The gene of *RB1* displays a wide spectrum of mutations, including small insertions/deletions (indels), large deletions/duplications, structural variations (SVs), and single nucleotide variations (SNVs) [6–8]. These mutations which are spanning 27 exons are distributed throughout the entire length of the *RB1*gene. No hotspots have been reported in this gene [9].

The incidence of retinoblastoma is approximately 1 in 16,000–18,000 live births, regardless of sex, race, or geography [10, 11]. In the United States and Europe, incidence rate of RB is 2–5 cases per million children according to WHO [12]. In India, rates of RB incidence are 1.9–12.3 and 1.3–6.7per million in boys and girls, respectively [13]. Dimaras Helen et al. have estimated that approximate 8000 new cases are predicted each year in worldwide [4]. In China, there are about 1100–1500 new cases each year, but only 50% those children survive [14]. The disease-free survival rate of children with RB in developed countries is over 95% [15] while this is substantially lower, at 10–30% in developing countries [16, 17].

Six countries of Asia-Pacific region bear 42.6% global burden of RB (3452 of 8099 children):1486 children in India (18.3%), 1103 children in China (13.6%), 277 children in Indonesia (3.4%), 260 children in Pakistan (3.2%), 184 (2.3%) children in Bangladesh, 142 (1.8%) children in Philippines [18].

A heritable form and non-heritable form are two forms of the RB disease. Heritable RB accounts for 45% of all cases which 80% bilateral, 15% unilateral and 5% trilateral. Approximately 55% of cases are non-heritable RB that is always unilateral [19]. The mode of RB inheritance is autosomal dominant (AD) found in 10% of cases.

[20]. The majority of RB patients are sporadic and the only affected members of otherwise unaffected families. The disease is labeled “sporadic” if the family has no history of RB [21].

From presenting signs and clinical examination, diagnosis of RB is usually clear [22]. Leukocoria (white pupil) is the most common sign; Strabismus is the second most common sign when central vision is lost. Due to increased pressure, or non-infective orbital inflammation, advanced disease might present with enlarged cornea and iris colour change [4, 23].

The primary goal of management is to save the child’s life, followed by salvage of the eye and optimization of visual function. Many treatment options of RB are available and depend on the laterality and extent of RB disease [24]. The various treatment modalities for retinoblastoma includes enucleation of the eye, external beam radiotherapy, brachytherapy, thermotherapy, laser photocoagulation, cryotherapy,systemic chemotherapy, intra-arterial chemotherapy, nanoparticulate chemotherapy and chemoreduction [4]. When children with RB have symptoms, such as leukokoria and strabismus, it is late for these children to have best time for cure. Delayed diagnosis results in incurable invasion of the optic nerve and brain and metastases elsewhere in the body. However, if noticed early, prompt treatment can cure the cancer and save the eye(s) [4]. According to Intraocular International Retinoblastoma Classify (IIRC) which divides retinoblastomas into 5 groups (labeled A through E; Group A means small tumors while group E means large tumor and the eye have no chance to be saved),Children’s Hospital of Fudan University reported that about 75% children with RB were diagnosed at group D and E of IIRC when these children saw doctor for the first time [25, 26].

In the management of RB, genetic testing and counseling are extremely important [27]. Heritability form of RB can be identified by genetic testing of the proband [28]. But, genetic testing has not been carried out in Yunnan whose permanent population was 47,368,000, including 51.48% male population and 48.52% female population, 17.79% children and 62.21% adults, 66.43%Han population and 33.57% minority population according to the 1% population sample survey by Yunnan Bureau of Statistics (http://stats.yn.gov.cn/).

Therefore, how to carry out genetic testing is an urgent problem in the RB clinical in developing countries. In this paper, the genes of RB family in Yunnan were studied in order to obtain genetic forms of RB by genetic testing of the probands and then give counseling to these families. We use targeted next generation sequencing (NGS) to screen probands and then Sanger sequencing was used to identify variants in other members of pedigrees. In our study, nine mutations were found and four were novel mutations.

## Methods

### Subjects

The non-consanguineous families from the Yun-Gui Plateau was recruited by the Children’s Hospital of Kunming Medical University for genetic diagnosis. In these families, the children suffered from RB, but the parents and other members were normal. This study was approved by the Ethics Committee of the Children’s Hospital of Kunming Medical University; in accordance with the principles of the Declaration of Helsinki written informed consent was obtained from the participants or their guardians. The age range of the patients, in which we obtained parental consent, is from 6 to 117 months (36.50 ± 38.61). In this study, the normal control includes 100 individuals (55 males and 45 females) aged between 5 and 35 years old without associated hereditary diseases.

### Clinical evaluations

All clinical and physical examinations were conducted in the Children’s Hospital of Kunming Medical University in Kunming. CT and ultrasound imaging were performed in these probands.

### Targeted NGS and variant analysis

Two-millilitre peripheral blood samples were collected from the probands, siblings and their parents in tubes containing 0.2 M EDTA. Using the QIAamp DNA blood extraction kit (TIANGEN, Beijing, China), DNA was extracted from the venous blood of each subject. According to the manufacturer’s protocol (MyGenostics, Inc., Beijing, China), 3 micrograms of genomic DNA was fragmented by Covaris 32, and the 3′ end of each DNA fragment was A-tailed. Then, Illumina adapters were ligated to these fragments. We aimed for a 350–400 base-pair product, and all samples were checked with Nanodrop 2000 or Qubit systems to determine if they represented a captured library.

Each resulting captured library was loaded on an Illumina MiSeq 2000 sequencing platform, and the sequences were determined to ensure that each sample met the desired average sequencing coverage.

### Mutation analysis

Using Bcl2Fastq software (Bcl2Fastq 2.18.0.12, Illumina, Inc.), raw image files were processed for base calling and raw data generation. In addition, to get a quality score ≥ 20, low-quality variations were filtered out. Then, Short Oligonucleotide Analysis Package (SOAP) aligner software (SOAP2.21, soap.genomics.org.cn/soapsnp.html) was used to align the clean reads to the reference human genome (UCSC hg19, http://genome.ucsc.edu/). Polymerase chain reaction (PCR) duplicates were removed by the Picard programme [29, 30] .The single nucleotide polymorphisms (SNPs) were determined by the SOAPsnp programme [31]. The reads were realigned by Burrows-Wheeler Aligner (BWA) software 0.7.15, and the deletions and insertions (InDels) were detected by Genome Analysis Toolkit software 3.7. In addition, the identified indel SNPs were annotated using the Exome-assistant programme (http://122.228.158.106/exomeassistant). To determine their pathogenicity, non-synonymous variants were evaluated by four algorithms, namely, PolyPhen (http://genetics.bwh.harvard.edu/pph2/), Protein Analysis Through Evolutionary Relationships (PANTHER, www.pantherdb.org), Sorting Intolerant from Tolerant [SIFT, (http://sift.jcvi.org/)] and Pathogenic Mutation Prediction (Pmut; http://mmb.pcb.ub.es/PMut/).

### Mutation validation

PCR and Sanger sequencing with an ABI3500 sequencer were used to confirm potential causative variants in this family. The sites of variation were identified to compare the DNA sequences with the corresponding GenBank (www.ncbi.nlm.nih.gov) reference sequences. The sequences of forward and reverse primers are presented in supplementary Table 1 and were used to confirm potential causative variants in this family.

Thermocycling conditions: An initial denaturation of 95 °C for 10 min, 35 cycles of denaturation at 94 °C for 30 s., annealing at 64 °C for 30 s, extension at 72 °C for 45 s and a final extension of 72 °C for 5 min. The sequences of forward and reverse primers are in supplementary Table 1.

## Results

### Clinical findings

Twelve patients with RB were selected for this study of targeted *RB1* sequencing from non-consanguineous Yunnan families. There are no pathogenic or likely pathogenic variants were detected in three probands with our approach while nine variants in nine families.

### The clinical findings of probands

**Fig. 1:**
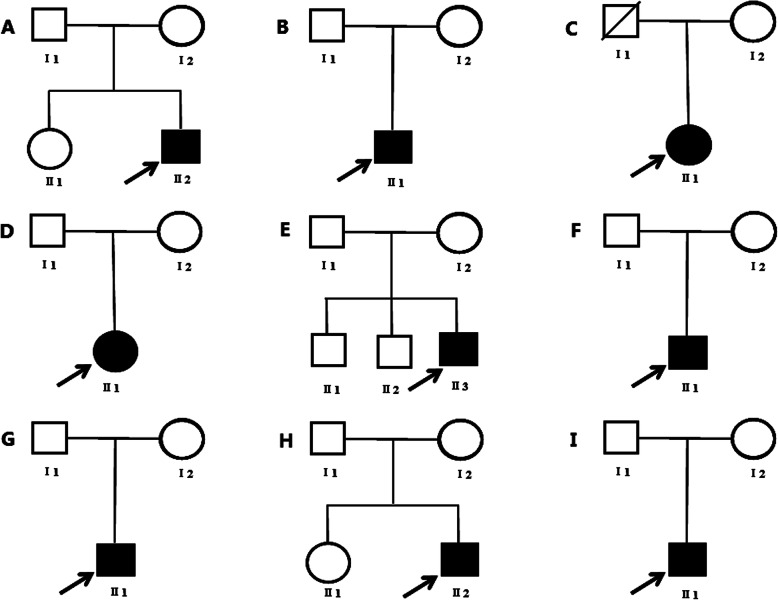
Pedigree of families with RB. Unaffected subjects are denoted as blank while affected subjects are represented with darkened symbols. The arrow indicates the proband. **a** .Pedigree of the family 1; **b**. Pedigree of the family 2; **c**. Pedigree of the family 3; **d**. Pedigree of the family 4;. **e**. Pedigree of the family 5; **f**. Pedigree of the family 6; **g**. Pedigree of the family 7; **h**. Pedigree of the family 8; **i**. Pedigree of the family 9

In these nine families,there are seven boys and two girls (See Fig. 1). Minority nationalities have Dai, Hani, Bai, Hui, Yi and Han. Mean age at diagnosis is 23 ± 9.9 months for unilateral and 11 ± 8.5 months for bilateral retinoblastoma in our study, respectively. Six probands were diagnosed with bilateral RB while three with the unilateral form. There are common sign leukocoria in probands of *families 1, 3, 4, 6, 7; in family 2,* left eye of the proband had been removed; the proband of 5 had strange reflection; the proband had blurred vision and shed tears in 8 and 9 (Table 1).

**Table 1:** Clinical features of patients with RB

Patient	Sex	Minority Nationalities	Age (Months)	Onset Age (Months)		Fundus Appearance	
				LE	RE	LE	RE
1	M	Dai	12	6	6	RB	RB
2	M	Hani	106	14 ^a^	17	–	RB
3	F	Bai	12	9	–	RB	–
4	F	Han	18	13	13	RB	RB
5	M	Hui	28	26	26	RB	RB
6	M	Yi	11	6	–	RB	–
7	M	Hani	60	54	–	RB	–
8	M	Han	6	5	5	RB	RB
9	M	Han	10	3	3	RB	RB

*Abbreviations*: *M* Male, *LE* Left eye, *RE* Right eye, Retinoblastoma (RB);The children of these families exhibited similar clinical features of RB. Ophthalmic examinations showed that the child in these families are affected by RB. ^a^ Removed at 15 months

### The clinical findings of parents

There was no congestion in the conjunctiva, no secretions in the conjunctival sac., and no tumor in both eyes. In both eyes (OU), the microscopy of slit lamp showed that the cornea is smooth and transparent, the thickness of corneal is normal, the depth of anterior chamber is normal, cornea is clear, iris texture is clear, pupil is isometric, lens is transparent, interstitial is clear and light emission exists.

### Targeted NGS

The mean read depth of coverage for each proband sample ranged from 52 to 66X, and the average throughput depth of the target region in each sample ranged from 90 to 99X. NGS produced reads from 32.8 to 56.12 million reads and the read length from 148 to 149 bp. The reads aligned to the human genome and mapped to the target region with a mean coverage from 99.8 to 99.9%. The SNPs and Indels are reported in supplementary Table 2.

### Identification of SNVs and InDels in children with RB patients

To detect SNVs and InDels, we analyzed blood samples of 6 bilateral patients and 3 familial unilateral patient and identified pathogenic variants in 8 patients and likely pathogenic variants in 1 patient. Four were novel and five were previously reported (Table 2). The spectrum of identified mutations includes 5 SNVs (1 nonsynonymous and 4 stop gain), one deletion (1 frameshift), and 3 splice site variants (splice).

**Table 2:** Variants identified by NGS in blood samples of RB chidren

No.	Locations on thechromosomes	Exon	Mutation		Pathogenicanalysis	Allele	Protein	Cosegregation infamily
1	chr13–48,942,673-48,942,675	11	c.1061_1062del	novel	pathogenic	het	p.R355Nfs*6	De novo
2	chr13–49,030,485	19	c.1960 G > C	known	Likely pathogenic	het	p.V654L	De novo
3	chr13–49,039,158	22	c.2236 G > T	novel	pathogenic	het	p.E746X	Heterozygous mother
4	chr13–49,047,526-49,047,530	24	c.2520 + 1_2520 + 4delGTGA	novel	pathogenic	het	splicing	De novo
5	chr13–48,955,538	17	c.1654 C > T	known	Pathogenic	het	p.R552X	De novo
6	chr13–49,027,168	18	c.1735 C > T	known	pathogenic	het	p.R579X	De novo
7	chr13–48,881,547	2	c.264 + 5G > A	novel	pathogenic	het	splicing	De novo
8	chr13–48,942,685	11	c.1072C > T	known	pathogenic	het	p.R358X	De novo
9	chr13–48,923,160	6	c.607 + 1G > A	known	pathogenic	het	splicing	De novo

^*^means termination

Eight of them were shown to be de novo, and remaining one was inherited from one of their mothers.

### Identification of pathogenic mutation

**Fig. 2:**
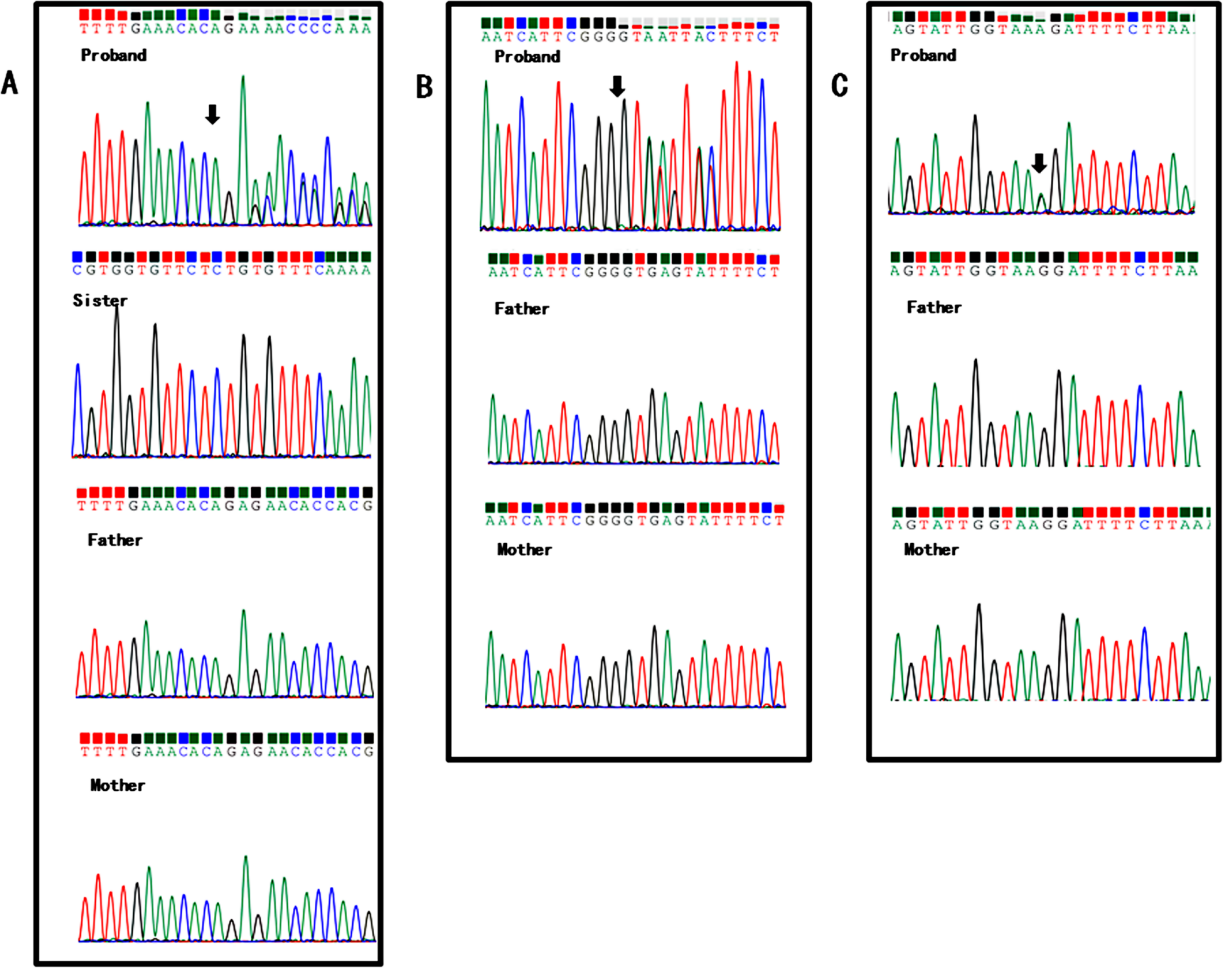
The variants of the probands. Arrows denote the mutations (the proband). Mutations of retinoblastoma identified by Sanger Sequencing in family 1, 4, 7. **a** The mutation of the family1; **b** The mutation of the family 4; **c** The mutation of the family 7

**Fig. 3:**
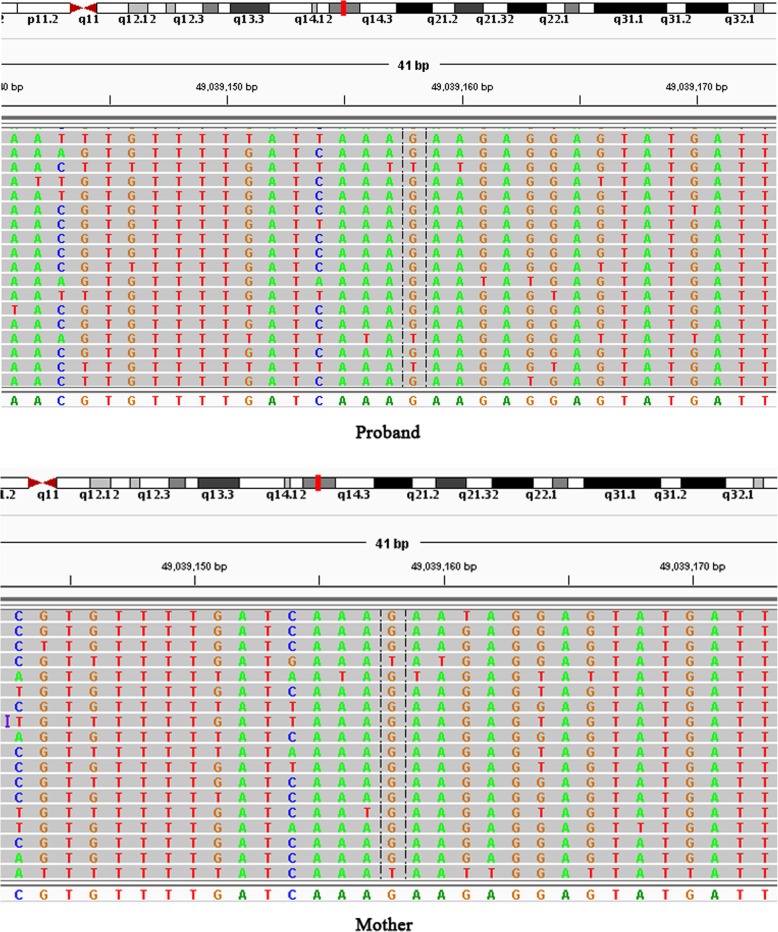
The variants of the probands in family 3

Sanger sequencing and cosegregation further confirmed all the pathogenic variants (See Figs. 2 and 3). We detected 9 mutations which 4 were novel and 5 were known (Table 2). Additionally, these four novel mutations were absent in 100 normal control individuals.

## Discussion

RB is the most common intraocular malignancy in childhood and presents in one or both eyes. Through presenting signs and clinical examination, diagnosis of RB is usually clear [22]. In our study, five probands had leukocoria; one proband had strange reflection; one proband’s left eye was enucleated; two had blurred vision. Mean age at diagnosis was 23 ± 9.9 months for unilateral and 11 ± 8.5 months for bilateral. According to Gene Reviews and AlAli et al. (Mean ageat diagnosis is 24 months for unilateral and 15 months for bilateral; mean age at diagnosis to be 27 months for unilateral and 15 months for bilateral, respectively), our mean age at diagnosis is not later than elsewhere in the world [24]. However, there no tumor in parents of these families.. And especially in family 3 and 7, the visions of the parents were normal.

Genetic testing of *RB1* is beneficial to provide counselling for families. In patients with RB, identification of gene alterations improves clinical management of patient and relatives at risk [32]. Here, we have used targeted NGS approach for the molecular analysis of Yunnan Children with RB, based on targeted gene enrichment and bioinformatics pipeline. We used in-house pipeline to successfully detect both pathogenic variants in RB patients. The average throughput depth of the target region in each sample ranged from 90-99X. These met the desired average sequencing coverage which was ensured to provide high quality bases for sensitive and efficient variant detection. To detect SNVs and InDels for all the samples, we developed an automatic in-house variant calling pipeline as freely available tools. The pathogenic SNVs and InDels were identified by stringent criteria, and 8 the pathogenic and 1 likely pathogenic variants were further confirmed by conventional methods and cosegregation with phenotype. Targeted mutation analysis is useful to study mutations in blood and can detect DNA variations. The *RB1* gene of seven families’ member are normal and only in family 3and 7, the unaffected mother of this family are heterozygous and the same to the proband. Seven proband’s genes are spontaneous mutation. Four mutations were newly discovered mutations and never reported before. With genetic testing, mutation profiles might be created to precisely screen mutations of relatives or subsequent generations in these families. Four families’ other children had no mutations by gene testing while other five families are one-child family.

However, no *RB1* mutations were found in the remaining 25% (3/12) of cases. This could be due to the fact that there are the weakness of study, like not doing cytogenetic studies such as chromosomal microarray (Identification of chromosome translocations or large gross deletions) and multiplex ligation-dependent probe amplification assays (MLPA identifies deletions or rearrangements of 1 or several exons, which account for 16% of all aberrations in RB1). This possibly results in 3 unsolved cases. The combined and cost-effective approach, such as method using a combination of direct sequencing and MLPA, were needed to accurately detect this 3 “negative cases” in next study.

Clearly, study of social determinants of health, such as health seeking behavior, perceptions of medical care, and sociocultural issues related to cancer inheritance would inform counselling approaches that meet the needs of families [33]. This study is helpful for the molecular diagnosis of RB in a comprehensive in Yunnan. These might provide molecular diagnosis to doctor for RB management because lack of genetic testing counseling, poor access to multidisciplinary retinoblastoma-specific health care and socioeconomic factors are one of the factors of higher mortality globally. For the probands with germlinemutation, the molecular diagnosis also provides counseling because the later life of patients with heritable RB displayed that these patients have a high life¬time risk of developing second primary malignancies, such as osteosarcomas, melanomas and soft-tissue sarcomas [34–36].

However, in this cohort, the small number of patients is not enough to establish a significant frequency reference and functional studies are necessary for assigning pathogenicity to these novel variants.

## Conclusion

Here, we reported that this approach with bioinformatics pipeline could detect variants including novel pathogenic variants. This comprehensive approach reduces the time and number of assays required for the detection of pathogenic variants by conventional methods. To the best of our knowledge, this is the first such study using targeted NGS approach to detect pathogenic variants in Yunnan children with RB. Overall, targeted NGS approach is becoming more feasible in clinical settings.

## Supplementary Information

**Additional file 1:** **Supplementary Table 1.**PCR primers for amplification. **Supplementary Table 2.** Coverage level through the target region for patients.

## Data Availability

The raw sequencing data of NGS are available in the NCBI’s Sequence Read Archive (SRA) with accession number PRJNA666751 (SRR12762344, SRR12762345, SRR12762346, SRR12762347) https://dataview.ncbi.nlm.nih.gov/object/PRJNA666751?reviewer=mlmsrbpj2u60r1ss0ijmkgmole”. The other data and materials are available from the corresponding author (JM) or first author (ZZ) upon reasonable request.

## Abbreviations

RB: Retinoblastoma
IIRC: Intraocular International Retinoblastoma Classify
NGS: Next generation sequencing
SOAP: Short Oligonucleotide Analysis Package
PCR: Polymerase chain reaction
SNPs: Single nucleotide polymorphisms
BWA: Burrows-Wheeler Aligner
InDels: Deletions and insertions
SIFT: Sorting Intolerant from Tolerant
SNVs: Single nucleotide variants
