# Supplementary material for: Next generation sequencing of RB1gene for the molecular diagnosis of ethnic minority with retinoblastoma in Yunnan
Source: BMC Med Genet. 2020 Nov 23;21:230. doi: 10.1186/s12881-020-01150-7 (PMC7682003; doi:10.1186/s12881-020-01150-7)
Supplement: Supplementary file 1 — Additional file 1: Supplementary Table 1.PCR primers for amplification. Supplementary Table 2. Coverage level through the target region for patients. [file 12881_2020_1150_MOESM1_ESM.docx]

Supplementary table 1 PCR primers for amplification

| **Patient** | **Exon** | **Forward primers** | **Reverse primers** |
| --- | --- | --- | --- |
| 1 | 11 | GCAGCAGCTGGGTCATCTAT | ACCACACCTGGCCTTCAATA |
| 2 | 19 | CCCAGCTTGCATTTAAATAGTCTG | GAAAGTAGAAGAAACATGATTTGAACC |
| 3 | 22 | AGGCTATTTCGTGCC | CACCTGGCCTTCAAT |
| 4 | 24 | TATGGGCAATGGCAGAATATG | TATGCCTGGATGAGGTGTTTG |
| 5 | 17 | AAATTGGAAGGCTATTTCCTATGAG | AGGTAGATGTTAAGAAACACCTCTCAC |
| 6 | 18 | TGCCACTGTCAATTGTGCC | GCAAATCCTAGGTGATTCAGTAGC |
| 7 | 2 | TGTTATGTGCAAACTATTGAAACAAG | AGCTCAGGCAATCCACCTG |
| 8 | 11 | GCAGCAGCTGGGTCATCTAT | ACCACACCTGGCCTTCAATA |
| 9 | 6 | CAGCTTCTCATGGTCAAGAATG | AAGCAGAGAATGAGGGAGGAG |

Supplementary table 2 Coverage level through the target region for patients

| Patient | **Reads**  **(M)** | **Read length**  **(bp)** | **Reads aligned to the human genome (Mb)** | **Reads mapped to the target region (Mb)** | **Mean**  **coverage** | **SNPs** | | **Indels** | |
| --- | --- | --- | --- | --- | --- | --- | --- | --- | --- |
|  |  |  |  |  |  | **Non-synonymous** | **Splice sites** | **Coding sequence** | **Splice sites** |
| 1 | 45.32 | 149 | 13535.97 | 13531.65 | 99.9% | 9662 | 835 | 751 | 255 |
| 2 | 39.43 | 149 | 11726.17 | 11715.66 | 99.8% | 12900 | 1084 | 972 | 313 |
| 3 | 56.12 | 148 | 16653.27 | 16631.34 | 99.8% | 9549 | 753 | 728 | 244 |
| 4 | 51.21 | 149 | 15252.03 | 15243.88 | 99.9% | 12766 | 1006 | 992 | 320 |
| 5 | 53.46 | 148 | 15863.68 | 15852.41 | 99.9% | 12681 | 983 | 970 | 314 |
| 6 | 36.7 | 148 | 10895.29 | 10888.65 | 99.8% | 12889 | 981 | 976 | 312 |
| 7 | 43.34 | 149 | 12876.37 | 12866.38 | 99.8% | 12736 | 1047 | 945 | 322 |
| 8 | 49.3 | 149 | 14654.79 | 14648.19 | 99.9% | 12870 | 971 | 939 | 287 |
| 9 | 32.8 | 149 | 9720.58 | 9708.46 | 99.8% | 12708 | 1024 | 935 | 308 |
